# How enoxaparin underdosing and sex contribute to achieving therapeutic anti-Xa levels

**DOI:** 10.3389/fphar.2024.1377232

**Published:** 2024-07-12

**Authors:** Alexander Tinchon, Joana Brait, Sascha Klee, Uwe Graichen, Christian Baumgartner, Oliver Friedrich, Elisabeth Freydl, Stefan Oberndorfer, Walter Struhal, Barbara Hain, Christoph Waiß, Dagmar Stoiber

**Affiliations:** ^1^ Karl Landsteiner University of Health Sciences, Krems, Austria; ^2^ Division of Neurology, University Hospital St. Pölten, St. Pölten, Austria; ^3^ Karl Landsteiner Institute of Clinical Neurology and Neuropsychology, University Hospital St. Pölten, St. Pölten, Austria; ^4^ Karl Landsteiner University of Health Sciences, Department of General Health Studies, Division of Biostatistics and Data Science, Krems, Austria; ^5^ Karl Landsteiner University of Health Sciences, Institute of Laboratory Medicine (Central Laboratory), University Hospital St. Pölten, St. Pölten, Austria; ^6^ Division of Neurology, University Hospital Tulln, Tulln, Austria; ^7^ Karl Landsteiner University of Health Sciences, Department of Pharmacology, Physiology and Microbiology, Division of Pharmacology, Krems, Austria

**Keywords:** anti-Xa, enoxaparin, underdosing, sex, gender, therapeutic, target range, anti-Xa levels

## Abstract

**Introduction:**

Anti-Xa serves as a clinical surrogate for assessing the efficacy and bleeding risk in patients treated with enoxaparin for thromboembolic events. Evidence from the literature and empirical observations suggest that patients are underdosed in clinical practice to avoid bleeding complications. This study aimed to investigate such underdosing of enoxaparin and its potential impact on achieving therapeutic anti-Xa levels.

**Methods:**

This multicentric, retrospective, observational study included patients with acute ischemic stroke due to atrial fibrillation. All patients received enoxaparin in the therapeutic setting with subsequent anti-Xa measurements. The one-sample, one-tailed Wilcoxon signed-rank test was used to identify a significant difference between the doses administered and the recommended daily dose. Logistic regression model analysis was performed to identify additional predictors affecting achievement of the therapeutic anti-Xa target range. Stepwise forward-backward selection with Akaike’s information criterion as metric was applied to refine the logistic regression model.

**Results:**

A total of 145 patients from the university hospitals of St. Pölten and Tulln in Lower Austria were included. The median daily enoxaparin dose administered was 1.23 mg/kg, resulting in an overall target range achievement rate of 66%. As compared to recommended therapeutic doses, significant underdosing of enoxaparin was evident in both participating centers (*p* < 0.001). The calculated threshold dose to achieve the therapeutic target range with a 90% probability was 1.5 mg/kg enoxaparin daily. Female sex was found to be a strong independent predictor of achieving a therapeutic target range (OR 9.44; 95% CI 3.40–30.05, *p* < 0.001).

**Conclusion:**

Despite the underdosing observed in both centers, therapeutic anti-Xa levels were achieved with lower than recommended doses of enoxaparin, and women required even lower doses than men. These findings warrant further confirmation by prospective studies.

## Highlights

- What is the current knowledge on the topic?
In certain patient cohorts, elevated anti-Xa levels have been observed with enoxaparin therapy, resulting in the need for dose adjustments to minimize the risk of bleeding. However, underdosing is noted beyond high-risk groups and there is limited documentation on the prevalence and consequences of this occurrence in routine clinical practice.
- What question did this study address?
The study aimed to identify real-world therapeutic underdosing of enoxaparin, its impact on achieving therapeutic anti-Xa levels, and relevant clinical variables influencing this target range achievement.
- What does this study add to our knowledge?
In clinical practice, enoxaparin is commonly underdosed for therapeutic purposes. Despite this underdosing, sufficient anti-Xa levels are often achieved, especially in women.
- How might this change clinical pharmacology or translational science?
Enoxaparin doses for sufficient therapeutic anticoagulation may be lower than recommended.

## Introduction

Low molecular weight heparins (LMWH) play a crucial role in human anticoagulation. They are characterized by their reduced molecular size compared to unfractionated heparin, with a molecular size of approximately 5,000 Da in contrast to 12,000–15,000 Da (Aguilar and Kleiman, 2000; Garcia et al., 2012). LMWH offer several advantages over unfractionated heparin, including an extended half-life, reduced susceptibility to heparin-induced thrombocytopenia, increased bioavailability, improved predictability to the anticoagulant dose-response, and the omission of routine laboratory monitoring (Aguilar and Kleiman, 2000; Garcia et al., 2012).

Predominantly, LMWH are used for the chemoprophylaxis of deep vein thrombosis and pulmonary embolism in immobilized patients (Zee et al., 2017). Enoxaparin is one of the most commonly prescribed agents for this purpose (Sherman et al., 2007; Rentsch et al., 2021; Taylor et al., 2021). Simultaneously, enoxaparin is utilized in therapeutic applications, primarily in the management of established venous thromboembolism or the treatment of low-to intermediate-risk pulmonary embolism (Leentjens et al., 2017; Robertson and Jones, 2017). Other examples include non-ST-segment-elevation myocardial infarction and cerebral venous thrombosis (Hulot et al., 2005; Yusuf et al., 2006; Ferro et al., 2017; Liu et al., 2021).

The assessment of the expected therapeutic effect of LMWH often relies on surrogate markers such as anti-Xa. This approach has the potential to identify patients at risk of suboptimal or excessive dosing, providing an opportunity for dose adjustments to mitigate the risk of recurrent thrombotic events or bleeding complications. The merits of this strategy remain a topic of debate within the medical literature, marked by conflicting findings (Dhillon et al., 2018; Karcutskie et al., 2018; van den Broek et al., 2022). However, there is sufficient evidence that anti-Xa levels outside the target range are associated with increased event or bleeding rates (Wu et al., 2020; May et al., 2022; John et al., 2023; Tischler et al., 2023).

Avoiding bleeding complications is the main reason for administering lower therapeutic doses in certain populations such as severely obese or renally insufficient patients (Hulot et al., 2005; Deal et al., 2011; Sacha et al., 2016; Jaspers et al., 2022). Dose reductions were also reported in a larger sample of patients with acute coronary syndrome and suspected increased bleeding risk (Montalescot et al., 2004). In ischemic stroke arising from atrial fibrillation, therapeutic doses of LMWH have often been used to prevent recurrent stroke while minimizing the risk of bleeding compared to conventional oral anticoagulants (IST, 1997; Berge et al., 2000). Potential fear of a possible overdose and subsequent bleeding may also led to underdosing in this population, which in turn could result in reduced anti-Xa levels. Thus, the primary objective was to assess the achievement of therapeutic anti-Xa levels based on the administered enoxaparin doses. Secondary objectives involved identifying clinical predictors and exploring optimal dose thresholds for achieving therapeutic anti-Xa levels.

## Materials and methods

### Patient selection

This study included patients from two large urban teaching hospitals in the federal state of Lower Austria who were admitted to a neurology department between 1 January 2013, and 28 February 2019. Inclusion criteria were age >18 years, diagnosis of acute ischemic stroke or transient ischemic attack (TIA) due to atrial fibrillation, and subsequent administration of enoxaparin in a therapeutic setting. Exclusion criteria included active bleeding, latelet counts below 100,000 per µl, congenital or acquired coagulopathies with a prothrombin time below 60 s, creatinine clearance (CrCl) below 30 mL/min, concomitant use of oral anticoagulants, improperly performed anti-Xa measurements, incomplete clinical records and pregnant women. Ethical approval was obtained from the local ethics committee (Ethics Committee of the Karl Landsteiner University of Health Sciences, No: 1016/2020) prior to study initiation.

### Data collection and analysis

Clinical data were extracted from electronic and handwritten medical records. A retrospective analysis was performed including the following variables: newly diagnosed ischemic stroke or TIA, sex, age, body weight, height, body mass index (BMI), enoxaparin doses administered, serum anti-Xa levels, prothrombin time, platelet count, CrCl, and clinical event rates, including recurrent stroke and bleeding. BMI categories were defined as < 18.5 for underweight, 18.5–25.0 for normal weight, 25.0–30.0 for overweight and >30.0 kg/m^2^ for obesity.

### Material

All patients received subcutaneous enoxaparin (*Lovenox* ^®^, Sanofi-Aventis GmbH, Vienna, Austria) daily every 12 h as prescribed by a physician (Lovenox prescribing information, 2022). Plasma anti-Xa levels were determined by a chromogenic assay (BIOPHEN Heparin LRT, Hyphen BioMed) after at least three previous consecutive administrations at peak levels 4 h after subcutaneous injection. Briefly, peripheral blood was collected by standard venipuncture into a 2-mL plastic tube containing 3.2% sodium citrate and centrifuged for 10 min with 1,865 g at room temperature. The plasma sample obtained was diluted with 0.9% NaCl (dilution factor 1:2). The chromogenic anti-Xa assay is based on the inhibition of a predetermined amount of factor Xa (added to each sample) by heparins or other factor Xa inhibitors in the presence of endogenous antithrombin (AT), followed by the cleavage of a factor Xa-specific chromogenic substrate (SXa-11) by the remaining factor Xa. During this reaction, the dye para-nitroaniline (pNA) is released from the chromogenic substrate, and correlates with the residual activity of factor Xa. The color development, measured at a wavelength of 405 nm, is thus inversely proportional to the anti-factor Xa activity of heparins or other factor Xa inhibitors in the sample. The therapeutic anti-Xa target range was set at 0.4–1.0 IU/mL.

### Study endpoints and statistical analysis

The primary objective was the achievement of a therapeutic target range at the actual doses of enoxaparin administered. Secondary objectives were the identification of clinical predictors and determination of enoxaparin threshold doses for achieving the therapeutic target range. In addition, the incidence of recurrent strokes and bleeding events was recorded. Recurrent stroke was defined as new cerebral ischemia detected by CT or MRI during the same hospitalization, categorized by size as < 1/3 (mild) and > 1/3 (moderate to severe) of the affected arterial supply area. Hemorrhages were classified by severity as mild to moderate and severe, and by location as intracerebral and extracerebral. Severe intracranial hemorrhage was defined by a clinically relevant mass effect, whereas severe extracranial hemorrhage was characterized by a clinically significant drop in hemoglobin requiring transfusion.

The threshold dose of enoxaparin required to achieve the therapeutic target range at 90% and 95%, respectively, was determined using a logistic regression model. Descriptive statistics were used to present patient demographics and clinical characteristics. The Shapiro-Wilk test was used to check the normal distribution of samples with continuous values. A one-sided, one-sample, Wilcoxon signed-rank test was used to check whether the median of a sample with continuous values was smaller than a given standard value. The Wilcoxon effect size was determined. A *p*-value <0.05 was considered significant. The Benjamini-Hochberg procedure was applied to correct for alpha inflation in multiple testing.

Multiple logistic regression techniques were used for exploratory analysis of the impact of various predictors. Thus, for this type of analysis, the sample size was estimated using the approach presented by Hsieh et al. (Hsieh et al., 1998). In this approach, the necessary sample number is estimated for a simple model and subsequently adjusted for the multiple logistic regression model, taking into account the variance inflation factor (VIF). In the simple model, we used the weight-based dose as the only predictor. We expected a strong influence on the outcome (achieving the anti-Xa target range); therefore we expected a log (odds) of at least 4. Furthermore, we assumed that the weight-based dose is nearly normally distributed. Within the process of data analysis, the VIF was used to test predictors for multicollinearity and predictors with a VIF >2.5 were removed from the model. Thus, for sample size estimation, a VIF = 2.5 or equivalently *R*^^2^ = 0.6 was assumed. For the sample size estimation, we assumed a type 1 error rate of alpha = 0.05 and a statistical power of (1–beta) = 0.8. Taking all these conditions and assumptions into account, a minimum sample number of 92 was necessary.

A logistic regression model was used to identify predictors that influence the achievement of the therapeutic anti-Xa target range. The following predictors were included in the initial logistic regression model: sex, age in years, weight in kg, BMI, weight-based daily dose, prothrombin time in %, baseline platelets in 10³ cells/μL, CrCl 60 ≥ mL/min, diagnosis (stroke, TIA), atrial fibrillation, and latency from start of LMWH to anti-Xa measurement in days. First, the multicollinearity of the predictors of the initial model was tested. The VIF was used as a metric. A VIF greater than 2.5 was found for the two predictors weight and BMI. As the predictor weight was considered more important in the context of the research question, BMI was removed from the list of predictors in the model. For further refinement of the logistic regression models, the approach of stepwise model selection with forward-backward search was used. The Akaike Information Criterion (AIC) was utilized as a metric to quantify the model quality (balancing model fitness and model complexity). After model refinement, sex, baseline platelets in 10³ cells/μL, diagnosis (stroke, TIA) and daily weight-based dose in mg/kg remained in the predictor list. To evaluate the effect size, coefficient of determination (Tjur’s R^2^) was calculated for the logistic regression model (Tjur, 2009). Statistical analysis was conducted using Gnu R software version 4.3.1.

## Results

### Patient screening and inclusion

**FIGURE 1:**
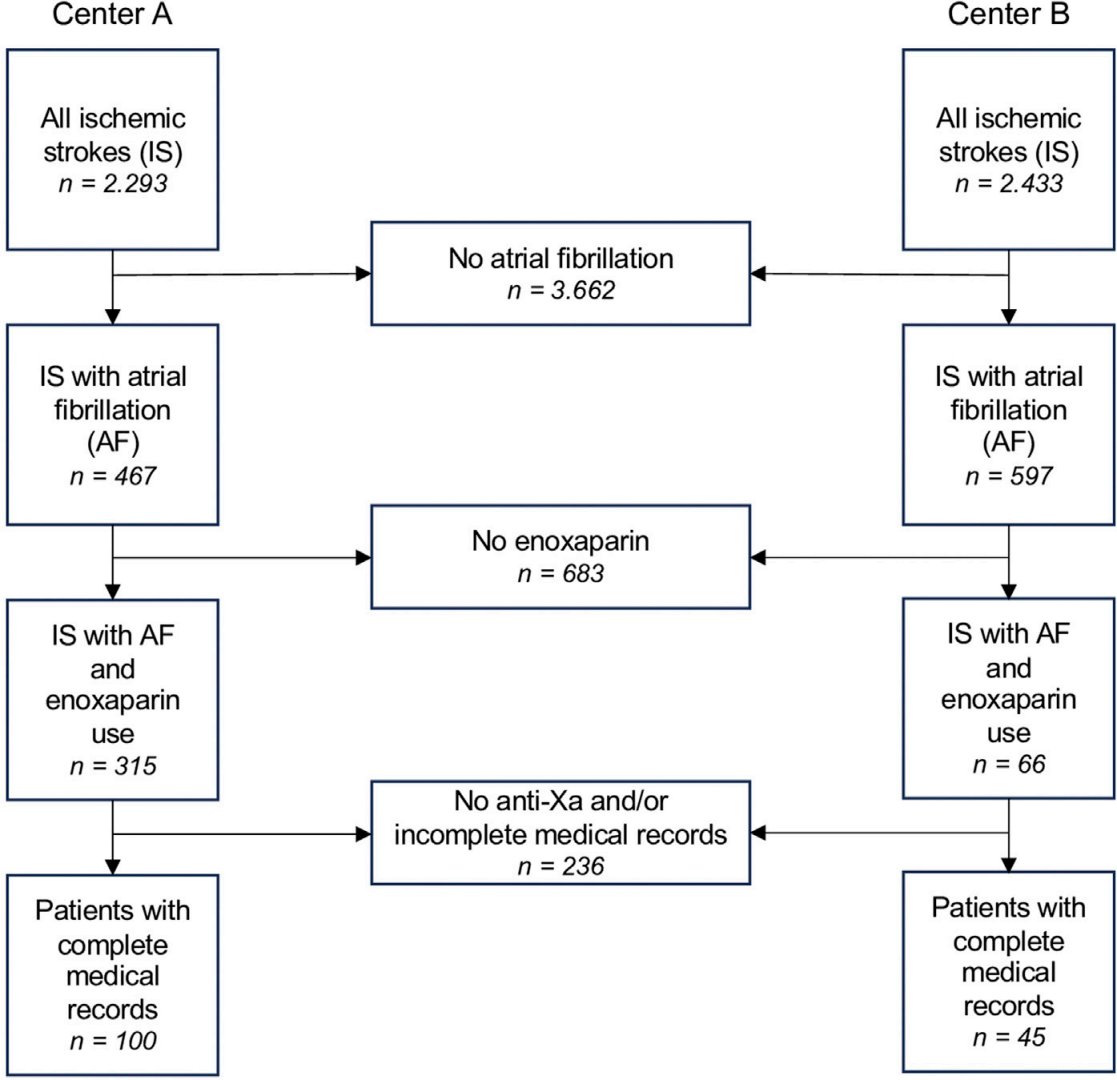
Patient selection flow chart for both centers (University Hospital St. Pölten = Center A, University Hospital Tulln = Center B). Abbreviations: IS, ischemic stroke; AF, atrial fibrillation.

Out of 4,726 initially screened patients diagnosed with newly diagnosed ischemic stroke, 1,064 had atrial fibrillation. In 683 of these cases, no enoxaparin or other LMWH was administered. Of the remaining 381 patients, 236 either had no anti-Xa measurement, no anti-Xa peak level or incomplete medical records, leaving 145 patients for the final analysis (Figure 1).

### Demographics

The patient cohort was predominantly male (59%), with a median age of 77 (68, 84) years, a median weight of 77 (68, 90) kg and a median BMI of 27.0 (24.2, 30.1) kg/m^2^. Stroke was diagnosed in 89% of patients, while 11% experienced TIA. Laboratory values, including prothrombin time and platelet count, were within normal reference ranges, with median values of 89 (81, 96)% and 219 (182, 256) 10^3^/µL, respectively. CrCl was greater than 60 mL/min in 74% and between 30 and 60 mL/min in 26% of patients. No statistically significant differences were observed in these parameters between the two centers (Table 1).

**TABLE 1:** Patient demographics.

		Center		
Characteristics^a^	Overall, n = 145^b^; Race: white (n = 145, 100%).	A, n = 100^b^	B, n = 45^b^	*p*-value^c^
*Sex*				0.4
Male	85 (59%)	56 (56%)	29 (64%)	
Female	60 (41%)	44 (44%)	16 (36%)	
Age in years	77 (68, 84)	76 (68, 84)	77 (69, 83)	0.8
Weight in kg	77 (68, 90)	75 (68, 86)	82 (68, 98)	0.4
BMI in kg/m^2^	27.0 (24.2, 30.1)	26.7 (24.2, 28.9)	28.1 (24.7, 32.3)	0.2
*BMI categories*				0.4
Underweight	0 (0%)	0 (0%)	0 (0%)	
Normal weight	42 (33%)	34 (34%)	8 (28%)	
Overweight	53 (41%)	43 (43%)	10 (34%)	
Obesity	34 (26%)	23 (23%)	11 (38%)	
Prothrombin time in %	89 (81, 96)	88 (80, 94)	91 (83, 103)	0.078
Baseline platelets in 10³ cells/μL	219 (182, 256)	217 (183, 250)	228 (182, 294)	0.4
CrCl < 60 mL/min	38 (26%)	27 (27%)	11 (24%)	0.8
*Diagnosis*				0.7
Stroke	129 (89%)	90 (90%)	39 (87%)	
TIA	16 (11%)	10 (10%)	6 (13%)	
Daily weight-based dose in mg/kg	1.23 (1.04, 1.60)	1.17 (1.01, 1.45)	1.50 (1.21, 1.83)	<0.001
Patients with doses <2 mg/kg daily^d^	138 (95%)	98 (98%)	40 (89%)	0.068
Anti-Xa IU/mL	0.52 (0.35, 0.67)	0.44 (0.34, 0.59)	0.70 (0.53, 0.87)	<0.001
Anti-Xa ≥ 0.4 IU/mL	96 (66%)	57 (57%)	39 (87%)	0.002

*Description of patient cohort.*

^a^ BMI: body mass index; CrCl: creatinine clearance; TIA: transient ischemic attack.

^b^ n (%): Median (IQR).

^c^ Benjamini-Hochberg correction for multiple testing.

^d^ Recommended therapeutic enoxaparin dose = 1 mg/kg body weight every 12 h.

The median enoxaparin dose administered was 1.23 (1.04, 1.60) mg/kg (daily dose administered in two single doses every 12 h), with a significant difference between the two centers (1.17 (1.01, 1.45) versus 1.50 (1.21, 1.83) mg/kg). Thus, anti-Xa levels also differed significantly, with values of 0.44 (0.34, 0.59) versus 0.70 (0.53, 0.87) IU/mL (Table 1).

Overall, 66% of patients achieved the therapeutic target range with the enoxaparin doses specified above. Due to the different dosing in the two centers, the achievement of the therapeutic target range also differed significantly at 57% versus 87% (Table 1).

**FIGURE 2:**
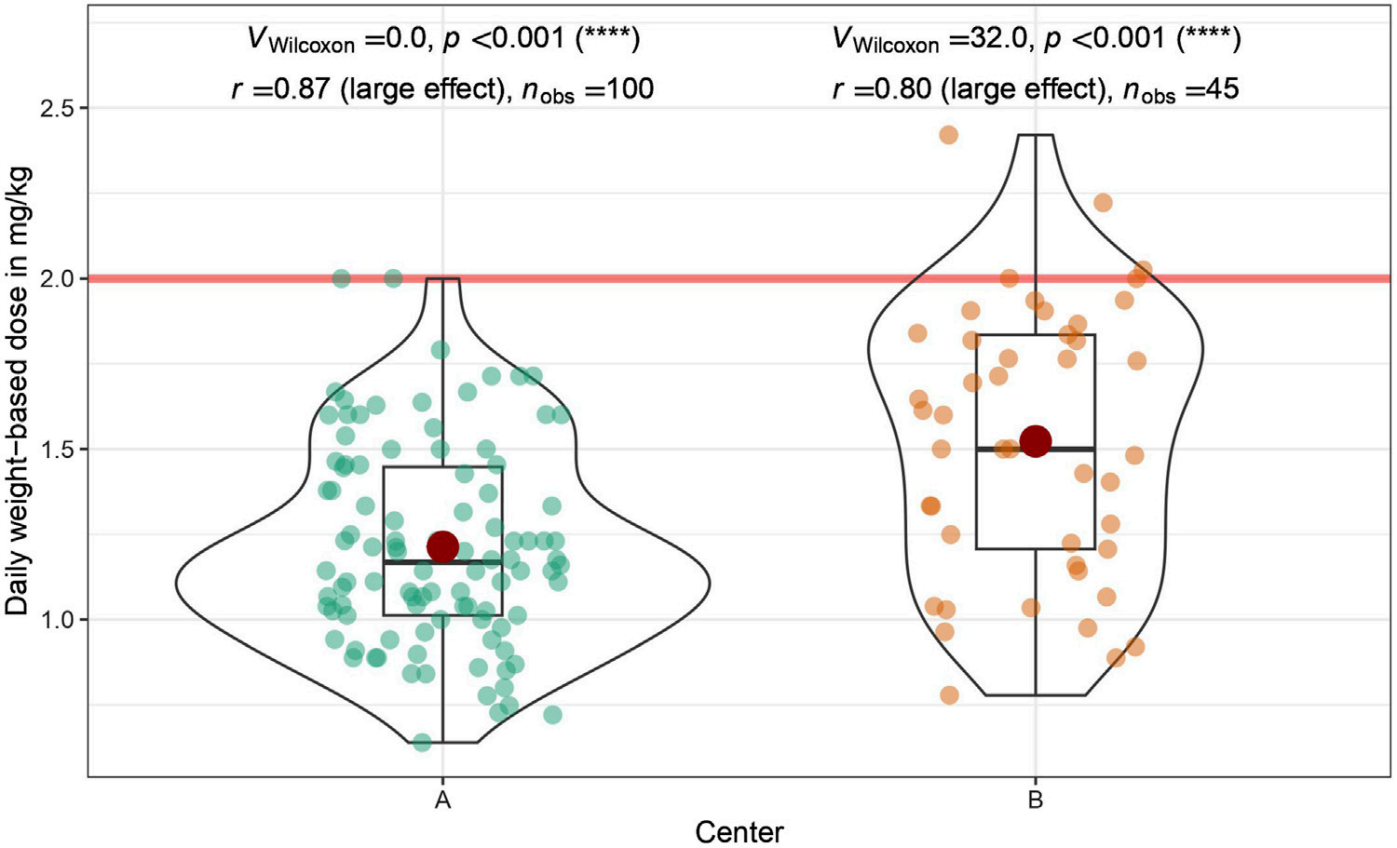
Daily weight-based dose of enoxaparin administered: Shown is a comparison between the two participating centers (University Hospital St. Pölten = Center A, University Hospital Tulln = Center B). The daily weight-based dose recommended by the drug manufacturer is marked by a red horizontal line. The doses administered were analyzed by a one-sided one-sample Wilcoxon signed rank test. In both centers, the dose administered is significantly lower than the recommended dose; Center **(A)** *p* < 0.001, *r* = 0.87; Center **(B)** *p* < 0.001, *r* = 0.80.

Despite significant differences in dosing, enoxaparin was administered well below the recommended daily dose of 2 mg/kg in both centers (Wilcoxon signed rank test: Center A: *p* < 0.001, *r* = 0.87; Center B: *p* < 0.001, *r* = 0.80; Figure 2).

### Predictors for achieving the therapeutic anti-Xa target range

Following refinement of the logistic regression models, sex, diagnosis, weight-based dose and baseline platelet count remained significant predictors. The coefficient of determination *R* ^2^_Tjur_, indicative of effect size, was calculated for the logistic regression model *R* ^2^_Tjur_ = 0.458. The odds of reaching the therapeutic target range were higher for women than for men (OR *9.44*, 95% CI: 3.40–30.05, *p* < 0.001, Table 2). Patients with TIA were more likely to achieve the therapeutic target range than patients with ischemic stroke (OR *5.36*, 95% CI: 1.12–33.07, *p* = 0.047, Table 2). Lower platelet counts were associated with a higher probability to reach the target range (OR *0.99*; 95% CI: 0.99–1.00, *p* = 0.047, Table 2).

**TABLE 2:** Clinical predictors for achieving a therapeutic target range.

	anti-Xa ≥ 0.4 IU/mL *R* ^2^_Tjur_ = 0.458		
Characteristics^a^	OR^b^	95% CI^b^	*p*-value^c^
*Sex*			
Male	—	—	
Female	9.439	3.396, 30.05	<0.001
Baseline platelets in 10³ cells/μL	0.992	0.985, 1.000	0.047
*Diagnosis*			
Stroke	—	—	
TIA	5.362	1.120, 33.07	0.047
Daily weight-based dose in mg/kg	371.5	53.29, 4,056	<0.001

*Predictors of the logistic regression model for achieving the therapeutic anti-Xa target range*. For the nominally scaled variables sex and diagnosis, the values *male* and *stroke* serve as *reference* for the odds ratio; in the table, they are marked by short horizontal dashes. The coefficient of determination *R* ^2^_Tjur_, which can be considered as an effect size, is given for each of the logistic regression models.

^a^ TIA: transient ischemic attack; *R* ^2^_Tjur_: Coefficient of determination.

^b^ OR: odds ratio; CI: Confidence Interval.

^c^ Benjamini-Hochberg correction for multiple testing.

### Calculated doses for achieving the therapeutic anti-Xa target range

**FIGURE 3:**
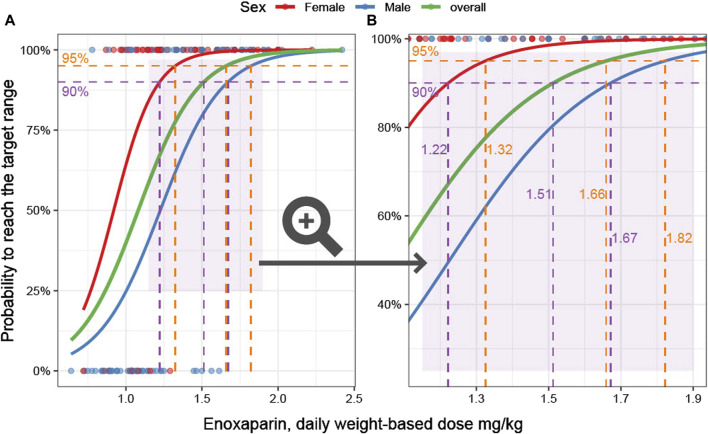
*Weight-based dose and probability to achieve therapeutic anti-Xa levels*. **(A)**: Overview. The probability to reach the anti-Xa target as a function of the predictor weight-based dose is the outcome parameter in this logistic regression model. The characteristics of female (red) and male (blue) patients are modeled separately. In addition, an overall model is given (green). The data underlying the models are visualized as dots. **(B)**: Zoomed-in view of the area marked in light purple in **(A)** is shown. The 90% and 95% probabilities are marked by dashed horizontal purple and orange lines, respectively. The corresponding weight-based doses are displayed on the vertical lines.

The calculated enoxaparin threshold doses required to reach the therapeutic range were found to be lower than the recommended dose of 2 mg/kg daily dose and exhibited differentiation according to sex. To achieve the target range with a 95% probability, the required daily dose was 1.66 mg/kg for the entire cohort, with it being 1.32 mg/kg for females and 1.82 mg/kg for males. In order to attain the target range with a 90% probability, the doses were 1.51 mg/kg for the entire cohort, 1.22 mg/kg for females, and 1.67 mg/kg for males (Figure 3).

### Clinical event and bleeding rates

During the initial hospitalization, three patients (2.1%) experienced recurrent strokes, and nine patients (6.2%) had bleeding events. Two of the strokes were mild, with one having a therapeutic anti-Xa level (0.85 IU/mL) and one having a subtherapeutic level (0.36 IU/mL). One moderate stroke was also linked to a subtherapeutic anti-Xa level (0.15 IU/mL). Of the nine bleeding events, eight were classified as mild to moderate, including three extracranial and six intracranial cases. One severe, non-fatal extracranial bleeding occurred with a supratherapeutic anti-Xa level (1.15 IU/mL). Of the mild to moderate hemorrhages, four had subtherapeutic anti-Xa levels (median 0.28 IU/mL; range 0.15–0.34 IU/mL), while the other four occurred within the therapeutic target range (median 0.61 IU/mL; range 0.47–0.96 IU/mL).

## Discussion

This study confirms empirical observations of enoxaparin underdosing in clinical practice.

It shows that achieving the desired therapeutic range often requires lower doses than recommended, especially in female patients.

In clinical practice, the conventional therapeutic enoxaparin regimen of 1 mg/kg every 12 h is often modified. Such adjustments can be attributed to a number of factors. For example, patients with severe obesity or impaired renal function have been shown to achieve therapeutic levels of enoxaparin at doses below conventional guidelines (Jaspers et al., 2022). Thus, unadjusted dosing carries the inherent risk of exposing patients to supratherapeutic levels of enoxaparin, thereby increasing their susceptibility to bleeding events (Barras et al., 2008).

A study conducted by Lee et al. on a cohort of overweight patients showed that 50% of them had supratherapeutic anti-Xa levels when given the standard dose of 1 mg/kg every 12 h (Lee et al., 2015). Additionally, Sacha et al. reported that 75% of severely obese patients were underdosed, receiving a median enoxaparin dosage of 0.89 mg/kg per dose (Sacha et al., 2016). An earlier case series by Deal et al. also described a reduced median therapeutic dose of 0.80 mg/kg per dose (Deal et al., 2011). However, these studies were hampered by a limited number of corresponding anti-Xa measurements, making it difficult to establish a definitive correlation with the dose administered.

Body weight may also have affected underdosing in our study population, considering that two-thirds were overweight. Notably, body weight did not appear to have a significant impact on reaching the therapeutic target range. This could be explained by the lower BMI of our patients compared with those in the previous studies. In addition, less obese people have been reported to have anti-Xa activity comparable to that of a non-overweight population (Sanderink et al., 2002). Renal function played a minor role in our sample, as the majority of patients exhibited normal CrCl, and individuals with severe renal insufficiency were excluded from the study.

Patient age may also contribute to underdosing. For example, in the ExTRACT-TIMI 25 trial, the therapeutic dose of enoxaparin was reduced to 0.75 mg/kg every 12 h in patients older than 75 years (Antman et al., 2006). Leri et al. specifically studied patients older than 65 years to describe the benefit of adjusted body-weight dosing versus standard dosing. Again, doses lower than the commonly recommended were used (Leri et al., 2009). Given the average age of 77 years in our sample, age may indeed have played a role in the dosing strategy adopted.

Overall, most of these deviations from the standard dosing regimens appear to be driven by a prevailing concern about the potential risk of iatrogenic bleeding due to an assumed overdose. As a result, even in prospective studies, clinical judgement often guides the decision to administer reduced doses of enoxaparin to patients perceived to be at increased risk of bleeding events (Montalescot et al., 2004). In our study population, concerns about intracranial hemorrhages may also have contributed to the cautious dosing approach (Hallevi et al., 2008).

Enoxaparin underdosing was observed in both participating centers, albeit to varying extents. As expected, this variability was also reflected in the resulting anti-Xa levels. Patients at the more pronounced underdosing Center A only marginally reached the therapeutic anti-Xa levels, whereas patients treated at the less underdosing Center B comfortably achieved anti-Xa levels well within the therapeutic target range. This leads us to question the required dose to consistently attain the therapeutic target range. It was explored that a daily dose of 1.5 mg/kg provided a 90% probability of achieving the therapeutic target range, a dose considerably lower than the standard recommended dose of 2 mg/kg daily. There was also a sex difference, with women needing a significantly lower dose of enoxaparin than men to achieve the therapeutic level with the same likelihood.

Evidence indicates that women attain comparatively higher anti-Xa levels in both prophylactic and therapeutic settings. A recent large retrospective study by Modi et al. reported that male trauma patients were more likely to have subprophylactic anti-Xa levels, while females were more prone to supraprophylactic levels (Modi et al., 2023). Similar findings were observed in burn patients by Cronin et al. and high-risk trauma patients by Farrar et al. (Cronin et al., 2019; Farrar et al., 2021).

These results are consistent with studies conducted in therapeutic settings. For instance, Leri et al. demonstrated that women were more likely to achieve the predefined therapeutic target range with weight-adjusted dosing, while Toss et al. reported higher anti-Xa activity in female patients during the acute treatment of unstable coronary artery disease (Toss et al., 1999; Leri et al., 2009). Oldgren et al. supported these findings in a larger sample, albeit with dalteparin and not enoxaparin, with both drugs differing in several clinical aspects such as antithrombotic potency, bleeding rates and bioavailability (Fareed et al., 1998; Oldgren et al., 2008).

In our study, women required lower doses of enoxaparin to attain the therapeutic range. Collinearity analysis ruled out interactions with other predictors, suggesting a genuine biological effect. The lower water content and reduced plasma volume in women could potentially concentrate hydrophilic substances, such as enoxaparin, in blood (Hakeam et al., 2020; Modi et al., 2023). Additionally, other sex-specific factors, including differences in muscle and adipose tissue distribution, pulmonary and renal function, and hormonal influences, could contribute to varying drug absorption, distribution, excretion, and interaction profiles (Franconi and Campesi, 2017).

An unexpected observation in our analysis concerned the higher probability of achieving a therapeutic anti-Xa range in patients experiencing TIA compared to those with ischemic strokes. TIA, as defined by the American Heart Association/American Stroke Association guidelines, represents a transient episode of neurological dysfunction attributed to focal cerebral, spinal cord, or retinal ischemia in the absence of acute infarction (Easton et al., 2009). Consequently, the primary distinction between TIA and ischemic strokes lies in the transient nature of symptoms. Nevertheless, both diseases share common features in pathophysiology. They are characterized by focal neurologic deficits attributable to impaired cerebral blood flow. Reports of differences in coagulation profiles between TIA and stroke are rare. For instance, Pelz et al. found increased fibrinogen levels in stroke patients, but these findings lost statistical significance after correction for multiple testing. Nonetheless, implementing clinical features and serum biomarkers have shown the potential to discriminate between TIA and stroke (Pelz et al., 2021).

Similarly, in the area of viscoelastometry, a technique to assess changes in blood viscosity by *in vitro* mechanical measurements, Bliden et al. observed a shorter time to initial clot formation in stroke patients than in TIA patients (Bliden et al., 2019). Ryu et al. reported shortened clot formation in stroke patients with worse functional outcomes at 3 months (Ryu et al., 2023). Both results suggest that measurable hypercoagulable coagulation profiles exist at least within the stroke population. Whether these observations support the results of our study must remain open at this time. However, given the small number of cases in this subgroup, incidental findings may also be considered.

Event rates in our sample generally align with those reported in other studies and were in the low percent range for recurrent stroke and bleeding events, rendering them clinically insignificant (Saliba et al., 2011; Lalama et al., 2015; Aleidan et al., 2020). Of note, both critical events corresponded to subtherapeutic or supratherapeutic anti-Xa levels. Although the bleeding rate was comparable with other studies, eight out of nine hemorrhages occurred in the therapeutic or even subtherapeutic target range. However, a larger number of cases will be required to test the plausibility of this observation.

Our study is subject to certain limitations. The retrospective design and number of cases may have biased the data. However, the results are based on a sample size estimation, which makes our number of cases seem sufficient. It should also be noted that these data from a large Austrian commuting area are not necessarily globally representative, particularly in terms of race and other demographic factors.

Due to underdosing, we barely found supratherapeutic anti-Xa levels. Therefore, we are unable to provide insight into the upper limits of the target ranges. It is plausible that the use of higher doses may have resulted in more cases with supratherapeutic anti-Xa levels. Nevertheless, supratherapeutic anti-Xa levels are more likely to be a concern in certain high-risk groups such as patients with severe renal insufficiency or massive obesity. Thus, a rather low number of supratherapeutic anti-Xa levels would have been expected in our sample, even at higher enoxaparin doses.

Our calculation of the enoxaparin dosage required to achieve the therapeutic target range with a 90% or 95% probability is limited by the absence of a recommended dose control for comparison. However, the fact that a considerable number of patients reached the therapeutic target range with notably lower doses provides a potential reference point that merits validation through prospective investigations.

It is important to note that our sample included mainly patients with atrial fibrillation and stroke in whom therapeutic use of LMWH is no longer indicated. Consequently, the generalizability of our results to other medical conditions is limited. However, we had a rigorously selected sample without severe renal dysfunction or morbid obesity. Therefore, similarity to other patient groups without high-risk constellations can be assumed with all due caution. These include, for example, patients with venous thromboembolism, cerebral sinus vein thrombosis or non-ST-segment-elevation myocardial infarction.

To evaluate enoxaparin therapy, we obtained anti-Xa peak levels. Emerging evidence suggests that trough levels provide greater accuracy, and this consideration should be taken into account in future study protocols. In addition, the observed sex differences are susceptible to the limitations associated with retrospective data analysis. Unaddressed confounders may have influenced our observations. Nevertheless, we included common clinical variables in our model and did not identify any confounding predictor.

In conclusion, despite significant underdosing, it was evident that enoxaparin doses below the recommended levels were sufficient to achieve the therapeutic target range. This observation raises the possibility of reassessing the current dose recommendations to potentially lower them, depending on the specific medical indication, as a measure to reduce the risk of bleeding. This is particularly important in high-risk groups such as patients with severe renal insufficiency or concomitant use of other anticoagulants.

In addition, it is prudent to consider sex-specific considerations in future prospective studies, particularly for women, as they may have a different sensitivity to therapeutic enoxaparin treatment compared to men.

## Data Availability

The original contributions presented in the study are included in the article/Supplementary Material, further inquiries can be directed to the corresponding author.

## Abbreviations

LMWH, Low molecular weight heparins; TIA, Transient ischemic attack; CrCl, Creatinine clearance; BMI, Body mass index; VIF, Variance Inflation Factor; AIC, Akaike Information Criterion.
